# The impact of comorbid type 2 diabetes on survival outcomes in patients with solid tumors treated with immune checkpoint inhibitors: a meta-analysis focusing on lung cancer

**DOI:** 10.3389/fendo.2026.1808976

**Published:** 2026-05-11

**Authors:** Linlin Fan, Weijie Xu, Qiang Zhang, Jin Li, Wenhui Bai

**Affiliations:** 1 Department of Comprehensive Ward, Sichuan Clinical Research Center for Cancer, Sichuan Cancer Hospital & Institute, Sichuan Cancer Center, University of Electronic Science and Technology of China, Chengdu, China; 2 Department of Medical Oncology, Jilin Cancer Hospital, Changchun, Jilin, China; 3 Department of Scientific Research, The Shapingba Hospital, Chongqing University (People’s Hospital of Shapingba District, Chongqing), Chongqing, China; 4 General Practice, The Shapingba Hospital, Chongqing University (People’s Hospital of Shapingba District, Chongqing), Chongqing, China

**Keywords:** cancer, immune checkpoint inhibitors, meta-analysis, prognosis, type 2 diabetes mellitus

## Abstract

**Background:**

Tumor patients with type 2 diabetes mellitus (T2DM) have a more immunosuppressive tumor microenvironment and weaker T-cell immune response to tumors within the tumor compared to non-T2DM patients when treated with immune checkpoint inhibitors (ICIs).In addition, high blood glucose levels may promote tumor immune escape. These factors may lead to a poor response to ICIs treatment in tumor patients with T2DM, affecting treatment prognosis. Although some studies have explored the association between tumor patients with T2DM and the prognosis of ICIs treatment, there is still controversy. Therefore, this study systematically evaluated the impact of T2DM on the prognosis of ICIs treatment in tumor patients through a meta-analysis, aiming to provide more accurate guidance for clinical practice and optimize the treatment strategy for tumor patients with T2DM.

**Methods:**

We systematically searched PubMed, Embase, Web of Science, CNKI, and Wanfang Database to collect studies published from the database establishment to January 2026 that investigated the association between tumor patients with T2DM and the prognosis of ICIs treatment. The Risk Of Bias In Non-randomized Studies - of Interventions (ROBINS-I) was used to evaluate the risk of bias. The pooled hazard ratio (HR) and 95% confidence interval (CI) were calculated to assess the association between tumor patients with T2DM and the prognosis of ICIs treatment. The primary outcomes included overall survival (OS) and progression-free survival (PFS). Meta-analysis was conducted using RevMan 5.3 software.

**Results:**

A total of six studies were included, involving 1,225 participants. The meta-analysis showed that patients with T2DM who received ICIs treatment had poorer OS (HR = 1.49, 95%CI:1.25-1.77, P < 0.00001) and PFS(HR = 1.38, 95%CI:1.04-1.83, P = 0.03).Subgroup analyses indicated that regardless of sample size (<200 vs >200) or type of survival analysis (univariate vs multivariate), patients with tumors and T2DM who received ICI treatment were consistently associated with poorer OS. Regarding PFS, a worse outcome was observed in T2DM patients when the sample size was less than 200 or when univariate analysis was applied. However, no significant statistical difference in PFS was found between non-T2DM and T2DM patients treated with ICIs when the sample size exceeded 200 or when multivariate analysis was performed.

**Conclusions:**

Based on the current limited evidence, this meta-analysis suggests that T2DM may be associated with poor OS in lung cancer patients treated with ICIs. However, due to the small number of included studies, limited sample size, inherent bias risks of the retrospective design, heterogeneity of tumor types, and the instability of PFS results. The conclusion of this study belongs to the ‘put forward hypothesis’ level and is not yet sufficient to support clinical practice recommendations. The current evidence cannot determine whether glycemic control can improve the efficacy of ICIs. Future studies need to verify this finding through large-sample, prospective cohort studies and clarify the independent impact of glycemic control levels on the efficacy of ICIs.

## Introduction

Malignant tumors are one of the major public health problems threatening the health of people worldwide (1, 2).The incidence rate and mortality rate are showing an upward trend year by year (1). Traditional radiotherapy, chemotherapy and targeted therapy methods have limitations in terms of efficacy and resistance when treating advanced tumors. In recent years, immune checkpoint inhibitors (ICIs), such as programmed death receptor 1 (PD-1), programmed death ligand 1 (PD-L1), and cytotoxic T lymphocyte-associated antigen 4 (CTLA-4) inhibitors, have been used to relieve the inhibition of tumor cells on the body’s immune system and re-activate anti-tumor immune responses (3). This has brought about a revolutionary breakthrough in the treatment of various malignant tumors, significantly improving the survival prognosis of patients with advanced cancer (4, 5). ICIs have demonstrated significant efficacy in a variety of solid tumors and hematological malignancies (6, 7). The emergence of ICIs has completely transformed the landscape of tumor treatment, bringing survival benefits to patients with various malignant tumors. However, the efficacy of ICIs treatment shows significant individual differences, and multiple baseline clinical characteristics and comorbidities have been proven to be the key factors influencing the benefits of ICIs.

Type 2 diabetes mellitus (T2DM) is one of the common chronic metabolic diseases characterized by high blood sugar levels worldwide, and its incidence is increasing year by year (8). In recent years, the association between T2DM and tumors has gradually attracted attention. Studies have shown that patients with T2DM have an increased risk of various malignant tumors, such as colorectal cancer, breast cancer, endometrial cancer, gallbladder cancer and hepatocellular carcinoma, etc (9, 10). Furthermore, T2DM is also associated with poor prognosis in cancer patients, including a decreased survival rate and an increased risk of disease progression (11–14). It is worth noting that T2DM, as an immunosuppressive state in itself, may affect the efficacy of ICIs (15). T2DM, as one of the most common metabolic diseases, has a relatively high prevalence among cancer patients (16). T2DM is closely related to the occurrence and development of tumors and may affect the tumor immune microenvironment and treatment response through multiple mechanisms.

Multiple studies (17–21) have explored the impact of T2DM on the prognosis of ICIs treatment, but the results are controversial. Some studies (18–20) have shown that the survival outcomes of patients with T2DM after receiving ICIs treatment are worse, while other studies (21, 22) have not found a significant correlation. This study aimed to comprehensively evaluate the impact of T2DM on the prognosis of tumor patients undergoing ICIs therapy through systematic review and meta-analysis. This study will provide evidence-based medical evidence for clinicians to formulate individualized immunotherapy strategies when dealing with tumor patients with T2DM.

## Methods

This study was conducted following the updated Preferred Reporting Items for Systematic Reviews and Meta-Analyses (PRISMA) guidelines (23–25).

### Data sources and search strategy

We systematically searched PubMed, Embase, Web of Science, CNKI and Wanfang Database to collect the literature published from the establishment of the databases to January 2026, which focused on the association between T2DM and the prognosis of ICIs treatment. The literature was comprehensively retrieved using a combination of MeSH terms and free words. Additionally, through reference tracing and citation tracking methods, relevant literature that might have been missed was supplemented for retrieval. The search terms were as follows: Type 2 diabetes mellitus,T2DM, diabetes mellitus type 2,immune checkpoint inhibitor, ICIs, ipilimumab,tremelimumab, CTLA-4 inhibitors, LAG-3 inhibitor, pelatlimab, PD-1 inhibitor, PD-L1 inhibitor,nivolumab,pembrolizumab,atezolizumab,durvalumab,overall survival,progression-free survival,etc.

### Inclusion and exclusion criteria

Studies were included if they met all the following criteria:

1. The participants were cancer patients;
2. Receive ICIs monotherapy or combination therapy;
3. The literature clearly reported the co-morbidity status of T2DM;
4. The literature provided survival outcome data [overall survival time (OS), progression-free survival time (PFS)], and sufficient data were provided to calculate the hazard ratio (HR) and its 95% confidence interval (CI);
5. The study type was a cohort study.

Studies were excluded if they met any of the following criteria:

1. Animal experiments or *in vitro* studies;
2. Case report or case series;
3. Studies without a control group or with incomplete prognosis data;
4. Re-published research;
5. Study without access to the full text.

### Data extraction

Two individuals independently used the Endnote software to conduct duplicate screening, data extraction, and cross-checking of the retrieved literature. If there was a disagreement between the two individuals, it was resolved through discussion. The extracted data information was as follows: 1) Basic information of the research: first author, publication year, research country, research type, sample size; 2) Patient characteristics: age, gender, tumor type, follow-up time; 3) Outcome indicators: OS, PFS, HR value and 95% CI; 4) Bias risk evaluation results.

### Risk of bias

The risk of bias in the included studies was evaluated using the Risk Of Bias In Non-randomized Studies - of Interventions (ROBINS-I). The risk of bias was assessed in seven domains: before the intervention (confounding bias, selection bias of study subjects), during the intervention (intervention classification bias), and after the intervention (deviation from the established intervention, missing data bias, outcome measurement bias, selective reporting of results). Finally, the bias of the studies was classified as low, moderate, high, or severe risk.

### Statistical analysis

Statistical analysis was conducted using RevMan 5.3 software. For survival data, HR and its 95% confidence interval were used as the main effect measure. Heterogeneity analysis was evaluated using the Cochran Q test and I² statistic to assess the degree of variation among studies. If P ≥ 0.10 and I² < 50%, there was no heterogeneity among the studies, and a fixed-effect model was used; if P < 0.10 and I² ≥ 50%, there was heterogeneity among the studies, and a random-effect model was used. Subgroup analysis was stratified by the following factors: (1) sample size (< 200 vs > 200); (2) type of survival analysis (univariate vs multivariate). Publication bias assessment was performed using funnel plots and Egger’s test. The funnel plot was used to visually display the relationship between the study effect size and sample size. Egger’s test was used to quantitatively assess the presence of publication bias. Sensitivity analysis evaluated the stability of the results by eliminating individual studies one by one, observing the changes in the combined effect size before and after elimination.

## Results

### Searching results

**Figure 1:**
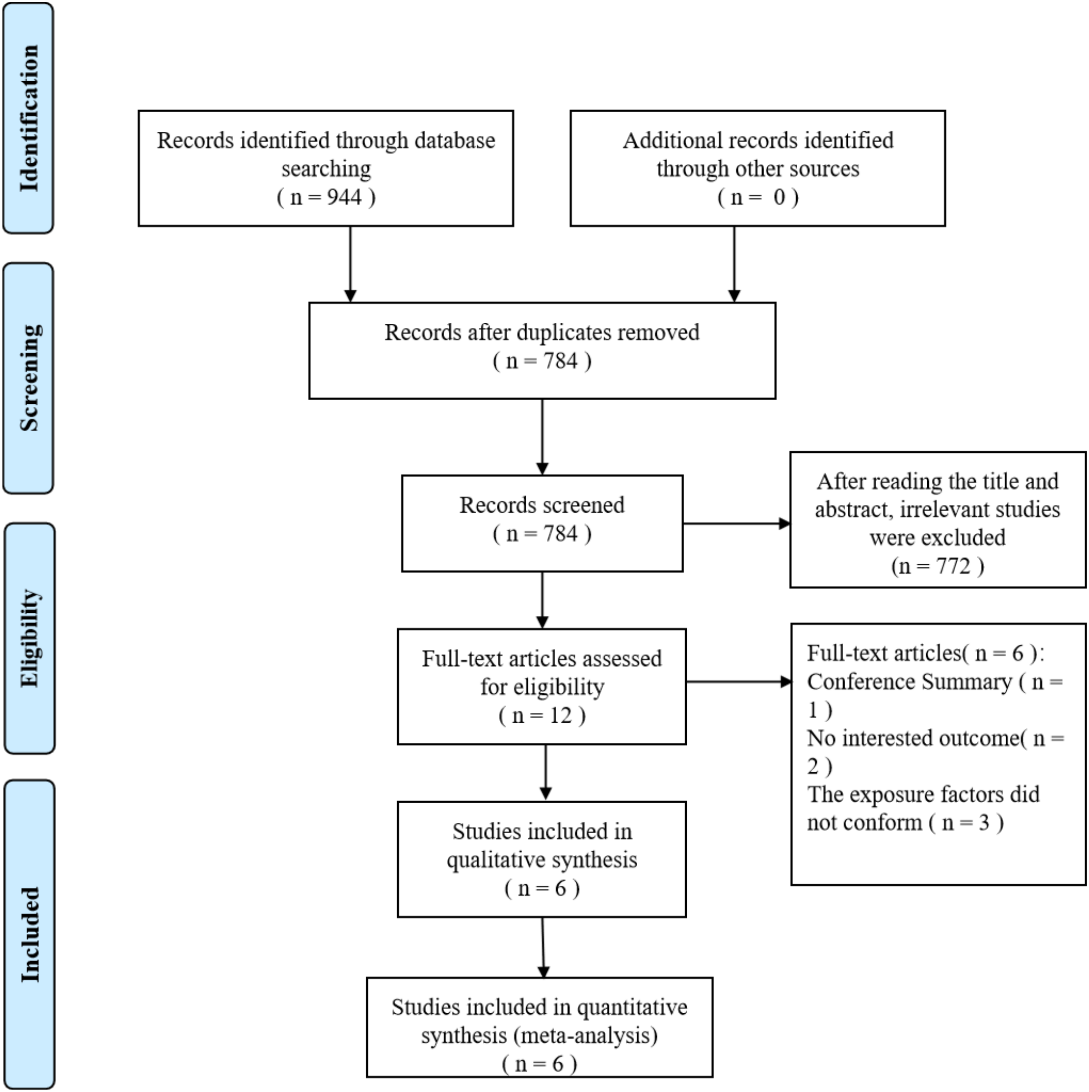
The flow diagram of this meta-analysis.

Through systematic search, 944 relevant papers were initially identified. After removing duplicates and screening based on titles and abstracts, 772 papers that did not meet the inclusion criteria were excluded, leaving 12 papers to be evaluated in full text. Eventually, six studies (17–22) were included (**Figure 1**).

### Characteristics of eligible studies

The basic characteristics included in the study were shown in **Table 1**. A total of six retrospective cohort studies were included, involving 1225 participants. These studies were conducted in multiple countries such as China, Israel, and Japan. The study period ranged from 2015 to 2024. The tumor types in five studies were lung cancer. One study was for melanoma. The sample sizes of these studies ranged from 88 to 441. All studies reported OS and PFS.

**Table 1:** Baseline characteristics of included studies.

Study, year	Country	Duration	Study design	Tumor type	Sample size	ICIs	Age (T2MD+/T2MD-)(mean)	Gender (male%, T2MD+/T2MD-)	Follow-up	Survival outcome	Analysis
Rong et al., 2025 (21)	China	From January 2019 to December 2023	Retrospective cohort study	NSCLC	441	NR	67.71/63.8	83.61%/76.75%	The median follow-up time was 41 months	OS,PFS	U
Hisanaga et al., 2021 (22)	Japan	From September 1, 2015 to July 31, 2018	Retrospective cohort study	Lung Cancer	88	Anti-PD-1 antibody	66.58/66.17	87.5%/79.7%	NR	OS,PFS	M
Jacobi et al., 2021 (17)	Israel	From February 2015 to April 2018	Retrospective cohort study	NSCLC	249	NR	69/66	68%/60%	The median follow-up time was 12.8 months	OS,PFS	U
Kato et al., 2025 (18)	Japan	From 2018 to 2024	Retrospective cohort study	NSCLC	115	NR	NR	89.2%/85.9%	The median follow-up time was 16.4 months	OS,PFS	U,M
Leshem et al., 2023 (19)	Israel	From January 2017 to July 2021,	Retrospective cohort study	NSCLC	203	Anti-PD-1 antibody	73/67	71%/61%	The median follow-up time was 26.9 months	OS,PFS	M
Mallardo et al., 2023 (20)	Several countries	NR	Retrospective cohort study	Melanoma	129	Anti-PD-1 antibody+LAG3 inhibitor	64/60	81%/80%	NR	OS,PFS	M

NR, not report; OS, overall survival; PFS, progression-free survival; NSCLC, non-small cell lung cancer; U, univariate; M, multivariate; NSCLC, Non Small Cell Lung Cancer; T2DM, type 2 diabetes mellitus; ICIs, immune checkpoint inhibitors; PD-1, programmed death receptor 1; PD-L1, programmed death ligand 1; LAG3, Lymphocyte Activation Gene-3.

### The result of the bias risk assessment

**Figure 2:**
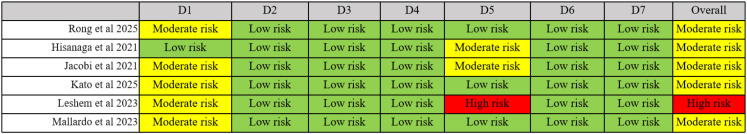
Risk assessment of bias.

This study used the ROBINS-I tool to assess the risk of bias for the six included retrospective cohort studies. The results showed that all the studies had a low risk of bias in terms of participant selection, intervention classification, intervention deviation, outcome measurement, and selective reporting. This indicated that the research design was relatively rigorous, and the patient grouping and outcome definition were clear and objective. However, in terms of controlling for confounding factors, except for the study by Hisanaga et al. (22), which achieved better control of confounding (low risk) through propensity score matching. The remaining five studies (17–20) all have moderate risks. Although these studies used multivariate regression to adjust for factors such as age, BMI, and stage, there may still be unmeasured confounding variables (such as differences in blood sugar control medications, duration of diabetes, severity of comorbidities, etc.) that could affect the accuracy of the efficacy comparison. In terms of data deficiency, the study by Leshem et al. (19) was rated as high-risk because it did not report key metabolic indicators such as blood glucose control levels (such as HbA1c). The studies conducted by Hisanaga et al. (22) and Jacobi et al. (17) were rated as having a moderate risk because for some cases, data on PD-L1 or HbA1c was missing and the handling methods were not adequately explained. Overall, the studies included in this review had a relatively low risk of bias in the core design phase, but there were certain limitations in terms of confounding control and data integrity, suggesting that caution should be exercised when interpreting the results. Future research should further strengthen the use of dynamic blood glucose monitoring and multi-factor matching designs. The results of the bias risk assessment of the included literature were shown in **Figure 2**.

### Meta-analysis

#### The association between type 2 diabetes and overall survival in cancer patients treated with immune checkpoint inhibitors

**Figure 3:**
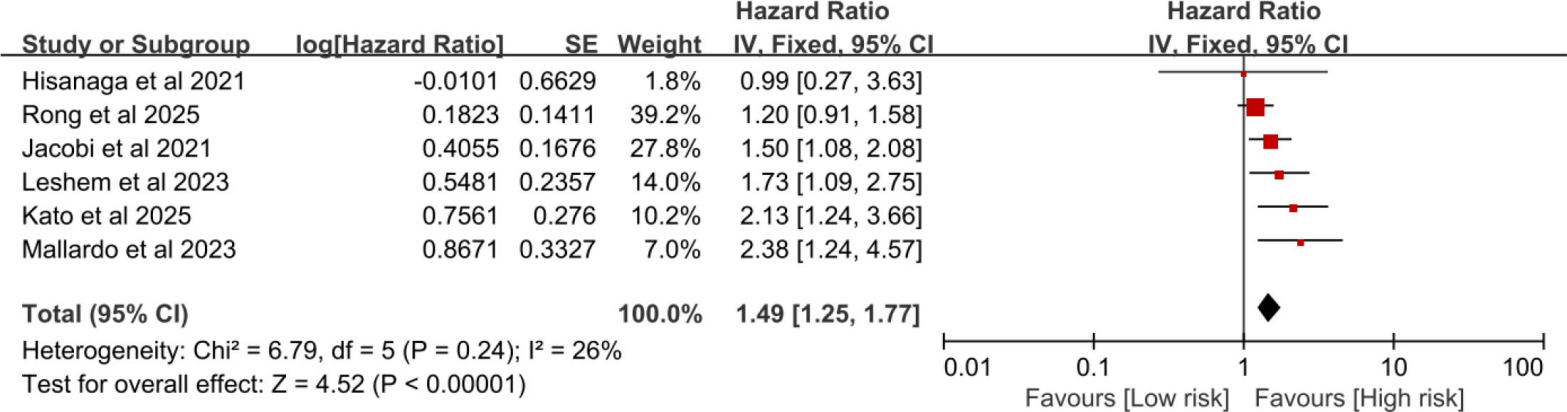
Pooled estimates of the association between type 2 diabetes and overall survival in cancer patients treated with immune checkpoint inhibitors.

**Figure 4:**
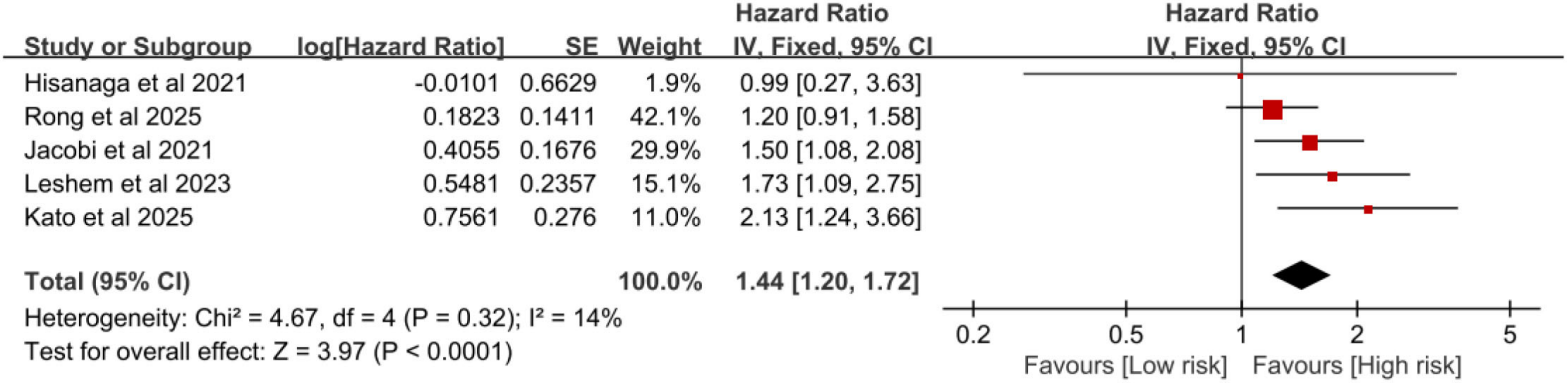
Pooled estimates of the association between type 2 diabetes and overall survival in lung cancer patients treated with immune checkpoint inhibitors.

Six studies reported the OS of tumor patients with T2DM who received ICIs treatment. The heterogeneity test was as follows: I^2^ = 26%, P = 0.24, and a fixed-effect model was used. The meta-analysis showed that patients with T2DM who received ICIs treatment had a poorer OS(HR = 1.49, 95%CI:1.25-1.77, P < 0.00001)(**Figure 3**). After excluding the study by Mallardo et al (20), the meta-analysis showed that lung cancer patients with T2DM who received ICIs treatment had a poorer OS(HR = 1.44, 95%CI:1.20-1.72, P < 0.0001) (**Figure 4**).

#### The association between type 2 diabetes and progression-free survival in cancer patients treated with immune checkpoint inhibitors

**Figure 5:**
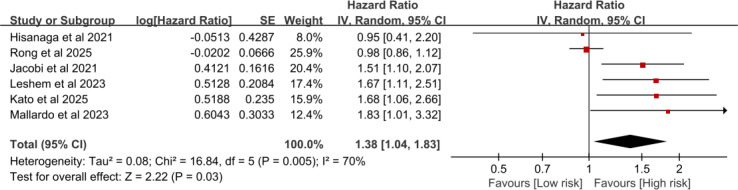
Pooled estimates of the association between type 2 diabetes and progression-free survival in cancer patients treated with immune checkpoint inhibitors.

**Figure 6:**
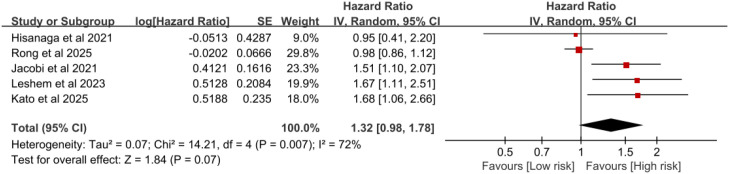
Pooled estimates of the association between type 2 diabetes and progression-free survival in lung cancer patients treated with immune checkpoint inhibitors.

Six studies reported the PFS of tumor patients with T2DM who received ICIs treatment. The heterogeneity test was as follows: I^2^ = 70%, P = 0.005, using the random effects model. The meta-analysis showed that patients with T2DM who received ICIs treatment had a poorer PFS (HR = 1.38, 95%CI:1.04-1.83, P = 0.03) (**Figure 5**). After excluding the study by Mallardo et al (20), the meta-analysis revealed that there was no significant statistical difference in PFS between lung cancer patients without T2DM and those with T2DM after receiving ICIs treatment (HR = 1.32, 95%CI:0.98-1.78, P = 0.07) (**Figure 6**).

### Subgroup analysis

We conducted subgroup analyses based on the sample size (< 200 vs > 200) and the type of survival analysis (univariate vs multivariate) (**Table 2**).

**Table 2:** Subgroup analysis summary results.

Subgroup analysis	The number of studies included	Heterogeneity		Model	Meta-analysis	
		P	I^2^		HR(95%CI)	P
OS						
Sample size						
< 200	3	0.49	0%	Fixed	**2.07(1.39, 3.07)**	**0.0003**
> 200	3	0.34	7%	Fixed	**1.38(1.14, 1.67)**	**0.001**
Analysis						
Univariate	3	0.16	46%	Fixed	**1.40(1.15, 1.71)**	**0.0008**
Multivariate	3	0.46	0%	Fixed	**1.83(1.27, 2.63)**	**0.001**
PFS						
Sample size						
< 200	3	0.43	0%	Fixed	**1.58(1.13, 2.20)**	**0.008**
> 200	3	0.005	81%	Random	1.31(0.90, 1.90)	0.16
Analysis						
Univariate	2	0.01	84%	Random	1.19(0.78, 1.81)	0.43
Multivariate	4	0.62	0%	Fixed	**1.61(1.25, 2.09)**	**0.0003**

Bold font indicated that the differences were statistically significant.

The subgroup analysis showed that regardless of sample size (< 200 vs > 200) and type of survival analysis (univariate vs multivariate), patients with tumors who had T2DM and received ICIs treatment were associated with a poorer OS.

The subgroup analysis showed that when the sample size was less than 200, patients with tumors who had T2DM and received ICIs treatment had a poorer PFS. However, when the sample size was greater than 200, there was no significant statistical difference in PFS between lung cancer patients without T2DM and those with T2DM who received ICIs treatment.

The subgroup analysis showed that when the survival data was univariate, patients with tumors who had T2DM and received ICIs treatment had a poorer PFS. However, when the survival data was multivariate, there was no significant statistical difference in PFS between cancer patients without T2DM and those with T2DM who received ICIs treatment.

### Sensitivity analysis

**Figure 7:**
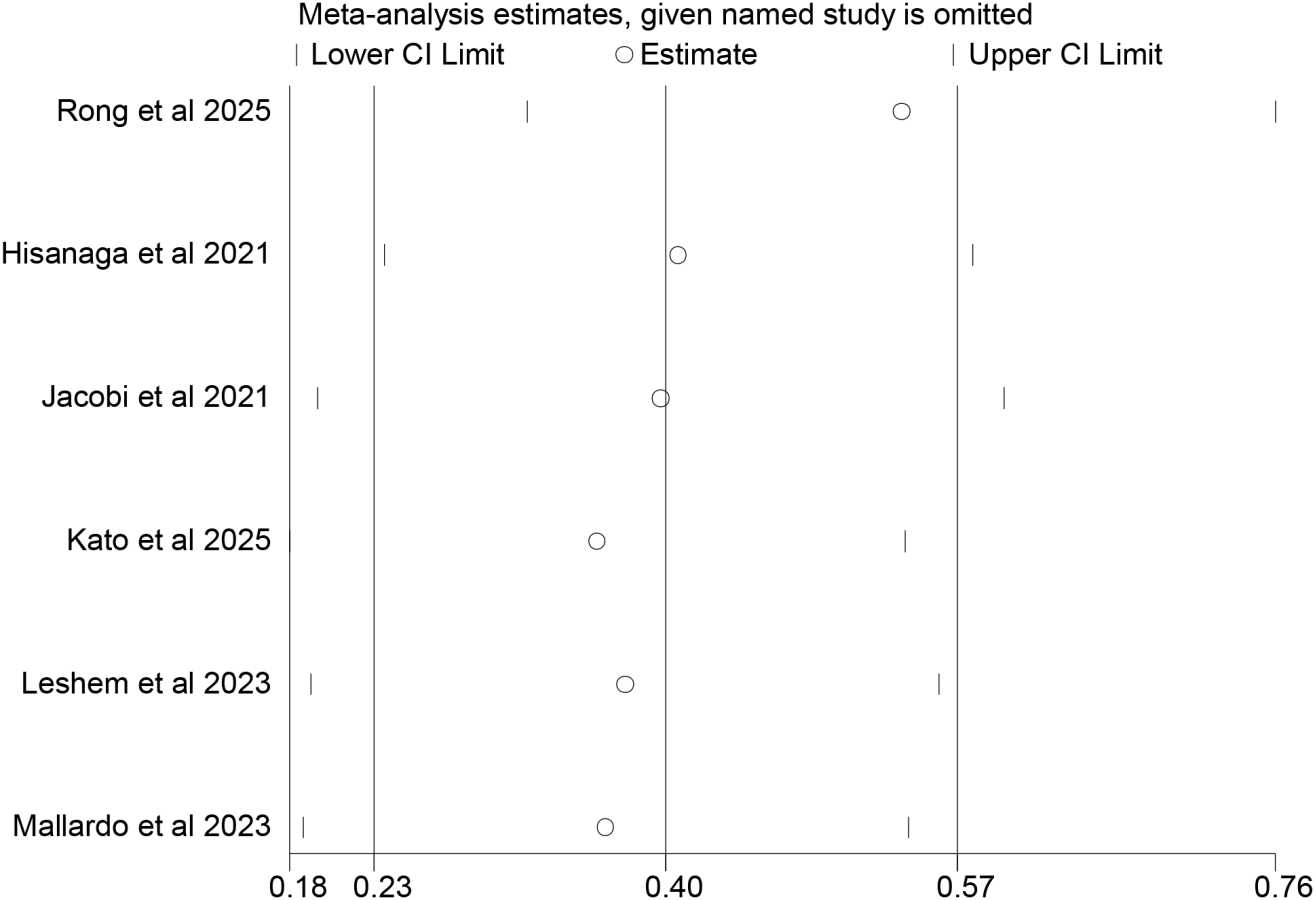
Sensitivity analysis of the association between type 2 diabetes and overall survival in cancer patients treated with immune checkpoint inhibitors.

**Figure 8:**
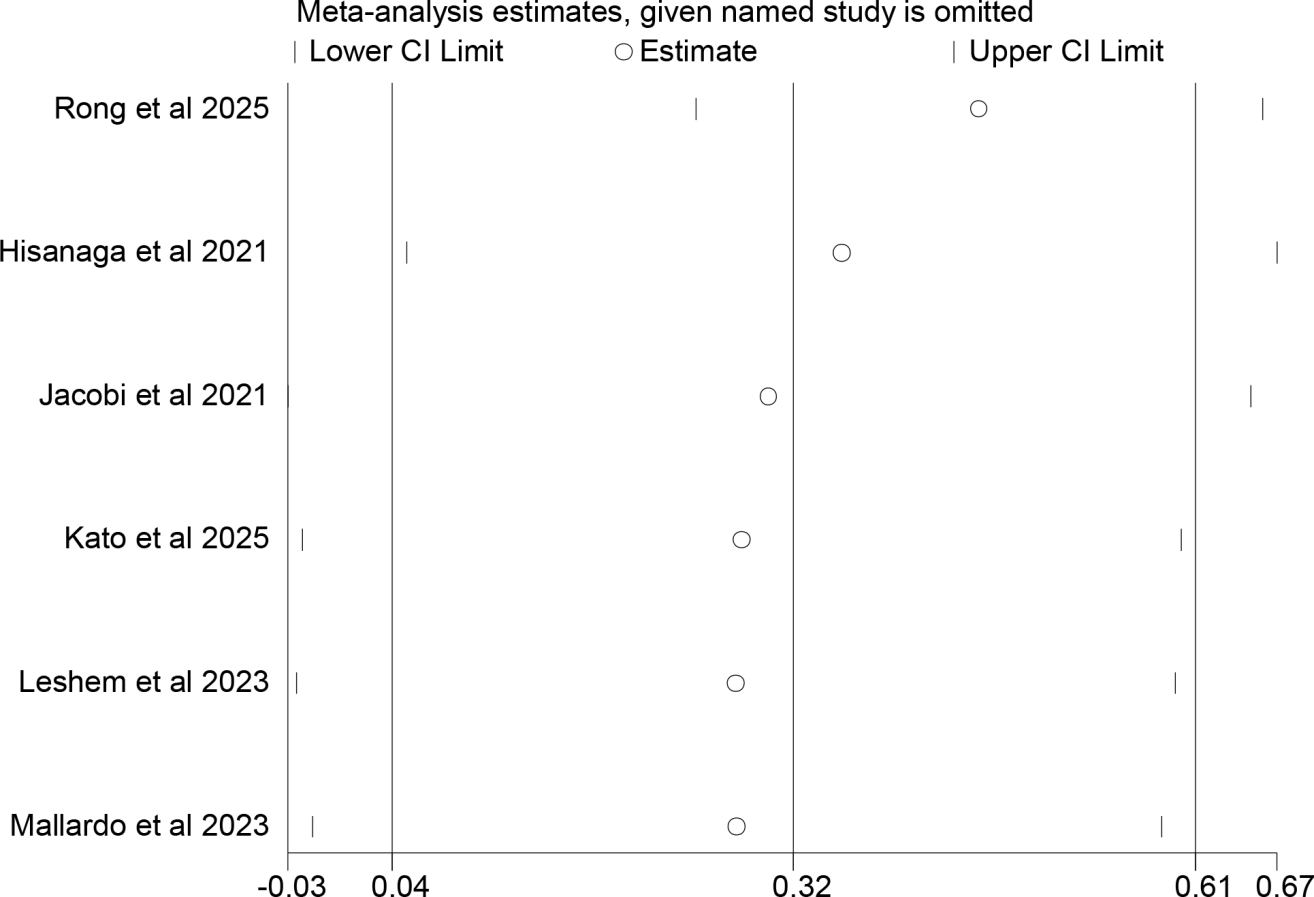
Sensitivity analysis of the association between type 2 diabetes and progression-free survival in cancer patients treated with immune checkpoint inhibitors.

A sensitivity analysis was conducted using the elimination-by-serial-method. The results showed that the meta-analysis results related to OS for tumor patients with T2DM who received ICIs treatment remained stable (**Figure 7**). After eliminating the studies by Jacobi et al (17), Leshem et al (19), Kato et al (18) and Mallardo et al (20) one by one, the meta-analysis showed that there was no significant statistical difference in PFS between tumor patients without T2DM and those with T2DM after receiving ICIs treatment (**Figure 8**). This indicated that the meta-analysis results of the association between T2DM and PFS after ICIs treatment were unstable.

### Publication bias

**Figure 9:**
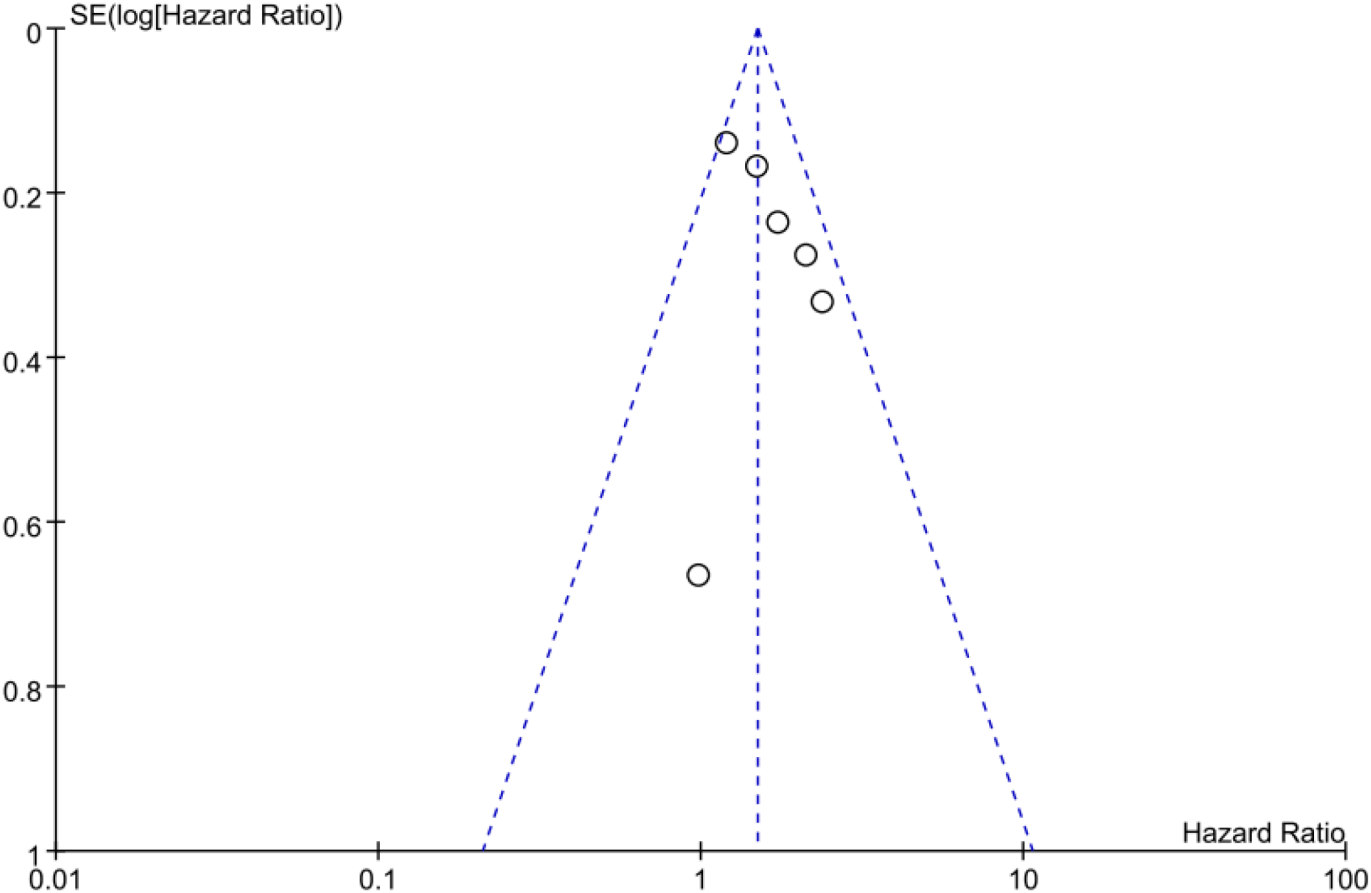
Funnel plot of the association between type 2 diabetes and overall survival in cancer patients treated with immune checkpoint inhibitors.

**Figure 10:**
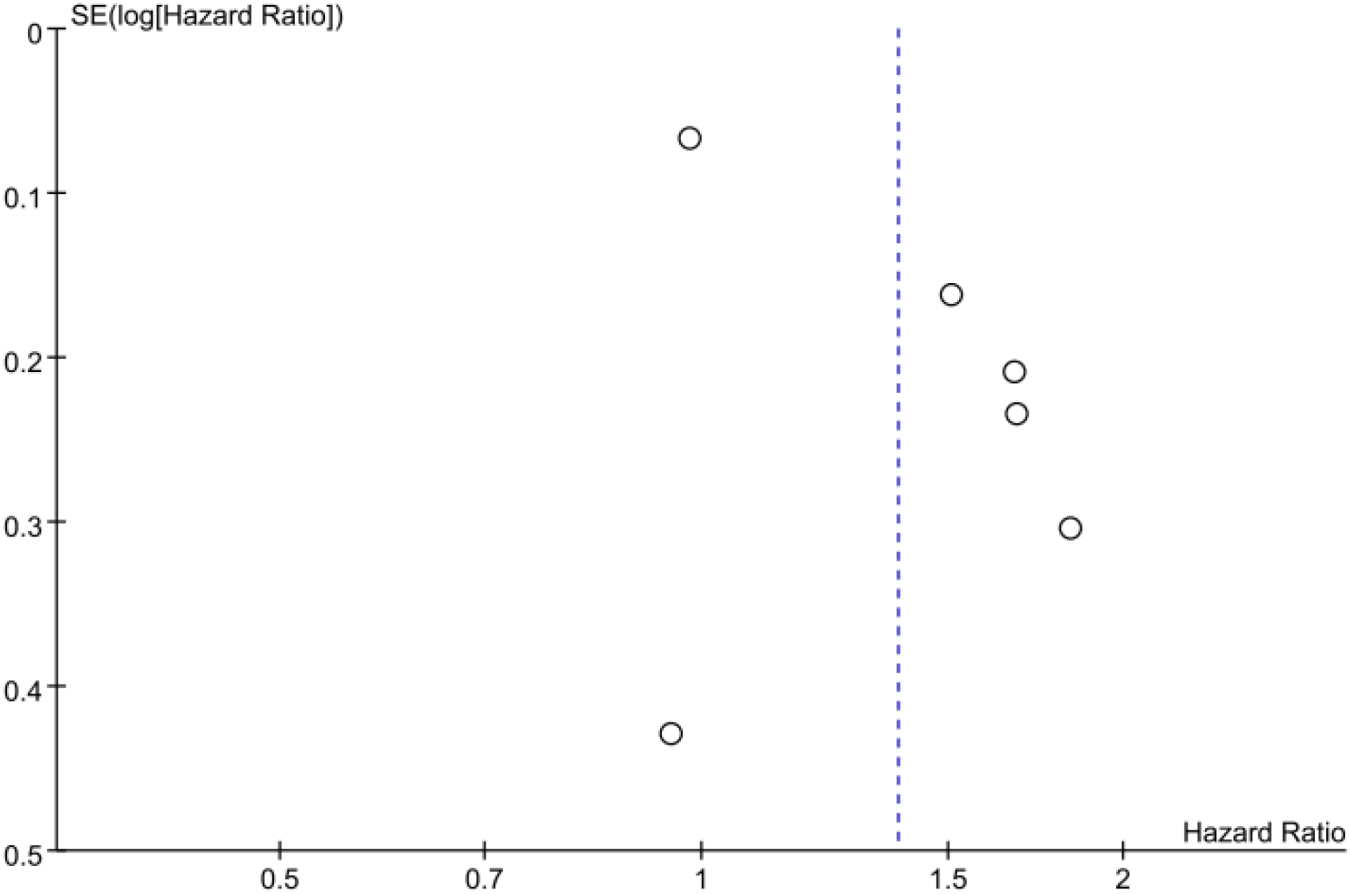
Funnel plot of the association between type 2 diabetes and progression-free survival in cancer patients treated with immune checkpoint inhibitors.

The inverted funnel plot shows that the scattered points on the left and right sides were not symmetrical, suggesting the possibility of publication bias (**Figures 9**, **10**). Egger’s test showed that P > 0.05, indicating no significant publication bias (OS: Egger’s test P = 0.264;PFS: Egger’s test P = 0.296).

## Discussion

This study conducted a meta-analysis to systematically evaluate the impact of T2DM comorbidity on the prognosis of ICIs treatment. The results showed that tumor patients with T2DM who received ICIs treatment had poorer OS and PFS. This finding is of great significance for clinical practice. When treating cancer patients, especially lung cancer patients, clinicians need to pay more attention to the T2DM comorbidity of the patients. For patients with T2DM, additional treatment strategies may need to be considered on the basis of the ICIs treatment plan to improve the prognosis, or other combined treatment methods may be explored to enhance the immune response.

The results of this meta-analysis were consistent with the conclusions of most of the included studies (18–20). Leshem et al. (19) reported that in patients with metastatic NSCLC, those with T2DM who received pembrolizumab experienced significantly shorter PFS and OS compared to those without T2DM.The studies conducted by Jacobi et al. (17) and Kato et al. (18)also supported the notion that T2DM was an independent negative prognostic factor for the efficacy of ICIs. These studies jointly indicated that a high blood sugar state was associated with a poorer immune treatment response. However, there were also inconsistent findings. The study conducted by Rong et al. (21) indicated that T2DM itself has no significant impact on the OS and PFS of patients with advanced NSCLC undergoing ICIs treatment. However, the survival period of the subgroup with poor fasting blood glucose control was significantly shorter. This suggests that the level of blood glucose control might be a more crucial factor than the diagnosis of T2DM itself. Similarly, Mallardo et al.’s research (20) in melanoma also found that patients with baseline high blood sugar or elevated blood sugar during treatment had a poorer prognosis. Currently, it is impossible to determine whether the association between T2DM and worse OS reflects a specific effect related to ICIs treatment, or simply because T2DM is known to be associated with the overall poor prognosis of cancer patients, regardless of the treatment method. All the included studies did not set up a non-ICIs control group, which makes it impossible to directly compare the prognostic impact of T2DM under different treatment strategies. Therefore, our study results cannot clearly attribute the survival disadvantage to the interaction between T2DM and ICIs. It is still possible that T2DM is just a marker of the overall poor prognosis of cancer patients, and the observed effect is independent of the type of treatment received. Hyperglycemia and the immunosuppressive tumor microenvironment related to T2DM may specifically hinder the efficacy of immunotherapy through certain reasonable biological pathways. However, these indirect mechanistic insights cannot replace direct comparative effectiveness studies. Future prospective studies should include both T2DM patients who received ICIs treatment and those who did not, to distinguish the treatment-related effects from the general prognostic effects. Before these data are available, caution should be exercised when interpreting our research results, and they should not be regarded as evidence of a specific interaction between T2DM and ICIs.

T2DM has a particularly significant impact on the prognosis of lung cancer patients (especially NSCLC), which may be related to the higher proportion of ICIs used among lung cancer patients. A cohort study on the association between metformin use and mortality in NSCLC patients receiving ICIs showed that in the cohort of NSCLC cancer patients who received ICIs, there was no statistically significant association between metformin use and cancer-specific or all-cause mortality (26). In the mouse model, the combination of metformin and anti-PD-1 treatment led to a reduction in tumor hypoxia induced by metformin, thereby enhancing the efficacy of PD-1 blockade (27). Among diabetic patients, those treated with metformin have a higher risk of death and a greater risk of disease progression/death (28). These findings are of great significance for clinical practice, as the global burden of T2DM is constantly increasing, and the clinical indications for ICI treatment are also expanding.

The chronic inflammation caused by high blood sugar levels and the immunosuppressive state of the tumor microenvironment are the main reasons for poor prognosis (28). T2DM, as a chronic inflammatory state, may affect the efficacy of ICIs through various mechanisms.T2DM, as a chronic inflammatory condition, may affect the efficacy of ICIs through various mechanisms. Firstly, the tumor microenvironment of T2DM patients exhibits immunosuppressive characteristics, including an increase in regulatory T cells (Tregs), an increase in the recruitment of myeloid-derived suppressor cells (MDSCs), and a polarization of M2-type macrophages (29–33). These changes lead to the impairment of tumor immune surveillance function, thereby affecting the efficacy of ICIs (34). High blood sugar can inhibit the proliferation and cytotoxic function of T cells, reduce the activity of natural killer (NK) cells, and affect the antigen-presenting function of dendritic cells (35–37). Furthermore, chronic inflammation is a significant characteristic of T2DM. The elevation of inflammatory factors such as IFN-γ and IL-6 may promote tumor progression and reduce the efficacy of immunotherapy (38).

The findings of this study are of great significance for clinical practice. The core value of this study lies in: 1) Systematically reviewing the existing evidence and revealing the preliminary signals that T2DM may affect the efficacy of ICIs. 2) Providing a reference for effect size in the design of future prospective studies (the combined HR of OS was approximately 1.49). 3) Suggesting that clinicians pay attention to the glycemic management of T2DM patients in real-world practice, but should not solely base on this conclusion to change the indication decisions for ICIs or refuse to use ICIs for T2DM patients. Any clinical decisions still need to be combined with the individual patient’s condition, the level of glycemic control, and more high-quality evidence. For patients with T2DM and tumors, the diabetes duration, blood glucose control level, and diabetes treatment drugs should be fully evaluated to formulate individualized ICIs treatment plans. Secondly, during ICIs treatment, blood glucose levels and the occurrence of immune checkpoint inhibitor-related adverse reactions should be closely monitored, and the treatment plan should be adjusted in a timely manner to improve the safety and effectiveness of the treatment. Moreover, this study also indicates the direction for future research. Future research should further explore the molecular mechanisms between T2DM and the therapeutic effect of ICIs to develop new treatment targets and strategies. Additionally, more clinical trials should be conducted to verify the impact of different diabetes treatment drugs on the prognosis of ICIs treatment, providing more solid evidence support for clinical practice.

Although this study reached meaningful conclusions through a meta-analysis, there were still some limitations. Firstly, the number of included studies was relatively small, only six, and the sample size was limited. This may have restricted the statistical power and generalizability of the results. Secondly, the main studies included in this study were retrospective cohort studies. The retrospective design cannot eliminate the interference of unmeasured confounding factors (such as blood glucose control levels, types of hypoglycemic drugs, duration of diabetes, etc.), and it cannot establish a causal relationship. Therefore, the conclusion of this Meta-analysis should be regarded as exploratory and hypothesis-generating evidence rather than a definitive basis for guiding clinical decisions. Clinicians should be cautious when interpreting it and avoid over-interpreting it as ‘T2DM patients should not use ICIs’ or ‘poor blood glucose control is an absolute contraindication for ICIs’. Thirdly, the results of PFS are not robust, there is significant heterogeneity (I² = 70%), and the sensitivity analysis shows that the combined effect loses statistical significance after excluding any one study. Therefore, the core finding of this study is limited to the association signal at the OS level and cannot draw the conclusion that ‘T2DM consistently worsens PFS’. More high-quality, large-sample prospective studies are needed to verify the impact of T2DM and related metabolic factors on the efficacy of ICIs (especially PFS). Fifth, since only one melanoma study was included, this conclusion cannot be generalized to melanoma and other tumor types. More studies targeting different tumor types are needed to verify the tumor type-specific impact of T2DM on the efficacy of ICIs. Finally, although we used ROBINS-I for bias risk assessment, we could not completely eliminate the influence of bias on the results.

## Conclusion

Based on the current limited evidence, this meta-analysis suggests that T2DM may be associated with poor OS in lung cancer patients treated with ICIs. However, due to the small number of included studies, limited sample size, inherent bias risks of the retrospective design, heterogeneity of tumor types, and the instability of PFS results. The conclusion of this study belongs to the ‘put forward hypothesis’ level and is not yet sufficient to support clinical practice recommendations. The current evidence cannot determine whether glycemic control can improve the efficacy of ICIs. Future studies need to verify this finding through large-sample, prospective cohort studies and clarify the independent impact of glycemic control levels on the efficacy of ICIs.

## Data Availability

The datasets presented in this study can be found in online repositories. The names of the repository/repositories and accession number(s) can be found in the article/supplementary material.
